# Effects of hyperandrogenism on metabolic abnormalities in patients with polycystic ovary syndrome: a meta-analysis

**DOI:** 10.1186/s12958-016-0203-8

**Published:** 2016-10-18

**Authors:** Rui Yang, Shuo Yang, Rong Li, Ping Liu, Jie Qiao, Yanwu Zhang

**Affiliations:** 1 Reproductive Medical Center, Peking University Third Hospital, No 49, North Huayuan Road, Haidian District, Beijing, 100191 China; 2 Institute of Medical Information (IMI) & Library, Chinese Academy of Medical Sciences and Peking Union Medical College, Beijing, 100730 China

**Keywords:** Hyperandrogenism, Metabolic disorder, PCOS, Meta-analysis

## Abstract

**Background:**

The study evaluated the effect of hyperandrogenism (HA) in polycystic ovary syndrome (PCOS) on metabolic parameters.

**Methods:**

We searched PubMed, EMBASE, Cochrane, Web of Science, Chinese Biomedical Database (CBM), China National Knowledge Infrastructure (CNKI), WanFang data and VIP for clinical observational studies. The study evaluated PCOS patients with or without HA on metabolic parameters was included. Prevalence of metabolic syndrome, indexes of insulin resistance (IR) including homeostasis model assessment IR index (HOMA-IR), incidence of IR, biomarkers of serum lipid metabolism such as total cholesterol (TC), triglyceride (TG), high density lipoprotein (HDL), and low density lipoprotein (LDL).

**Results:**

Of 4457 identified trials, 32 observational studies were included for the final analysis comprising 9556 female with PCOS. 6482 cases were having HA, and the others were negative. There were significant differences in the incidence of metabolic syndrome, HOMA-IR, rate of IR, TC level and HDL level between PCOS patients with or without HA, except for LDL level. No significant publication bias was found as *P* value of Egger’s test was 0.82.

**Conclusions:**

HA play an important role in metabolic disorders in PCOS patients. The incidence of metabolic syndrome, IR indexes, and most biomarkers of serum lipid metabolism were significantly different between patients with and without HA.

## Background

Polycystic ovary syndrome (PCOS) is a disease that mostly occurs in women of childbearing age. It is characterized by excessive androgen secretion and persistent anovulation. The incidence of PCOS is as high as 5 % ~ 10 % in women of childbearing age [1] (the prevalence is 5.61 % in Chinese women of childbearing age [2]), and it mainly manifests as oligomenorrhea/amenorrhea (O), oligoovulation/anovulation (O), and acne, etc., as well as obesity, hirsutism, and polycystic ovary (PCO), etc. Since 1990s, three diagnostic criteria have appeared for PCOS: Maryland diagnosis consensus developed by National Institutes of Health (NIH) in 1990, Rotterdam diagnosis criteria developed by European Society of Human Reproduction and Embryology (ESHRE) and American Society for Reproductive Medicine (ASRM) in 2003, and diagnosis criteria developed by Androgen Excess Society (AES) in 2006. Studies revealed different degrees of obesity, dyslipidemia, insulin resistance (IR), abnormal glucose metabolism, metabolic syndrome (MetS), and other metabolic abnormalities [2, 3] in PCOS patients. As one of the most important clinical features of PCOS, hyperandrogenism (HA) tends to cause IR, where the free androgen level is generally higher and the IR extent is also significantly aggravated in females with central obesity compared with normal control group. Different possible mechanisms were reported in various studies, which included the following: The androgen may directly or indirectly affect the glucose metabolism, thereby leading to HA. Second, the androgen may directly inhibit the effects of peripheral and intrahepatic insulin and cause HA. Furthermore, the androgen may increase the formation of free fatty acids, inhibit clearance of intrahepatic insulin, causing HA, thereby resulting in IR and metabolic abnormalities. This study aimed to identify the effect of the presence of HA on metabolic abnormalities in PCOS patients using systematic review and meta-analysis, thereby to provide reference for further in-depth studies, as well as to provide a basis for the treatment and prevention of long-term complications in PCOS patients.

## Methods

### Search strategy

Studies published between January 1980 and November 2014 were searched, where the computerized databases Medline, PubMed, Embase, Cochrane Library, and Web of Science were searched to identify eligible studies in English-language journals, while the computerized databases such as Chinese Biomedical Database, China National Knowledge Infrastructure (CNKI), Wanfang database, and VIP Information/Chinese Scientific Journals Database were searched for Chinese-language journals, and manual search or literature recall were supplemented. Keywords for the search included “polycystic ovary syndrome,” “hyperandrogenism,” “metabolic diseases,” and “metabolic syndrome,” etc.

### Inclusion and exclusion criteria

Inclusion criteria: (1) Observation studies including cohort studies, case–control studies, and cross-sectional studies; (2) PCOS patients with or without HA, or PCOS patients with different phenotypes, the diagnosis of which abided by the 2003 Rotterdam criteria or 2006 AES criteria; and (3) studies with primary outcomes including the incidence of MetS, insulin resistance indexes including homeostasis model assessment for insulin resistance (HOMA-IR) and incidence of IR, and lipid metabolism indexes including total cholesterol (TC), triglyceride (TG), high-density lipoprotein (HDL), and low-density lipoprotein (LDL).

Exclusion criteria:(1) Repeated and/or irrelevant literature, or literature with incomplete information; conference abstracts without detailed contents; academic dissertation; and literature review; (2) control group, or any unreasonable design, inexactor contradictory experimental results; (3) studies not stating clear diagnostic criteria for PCOS or adopting 1990 diagnostic criteria for PCOS issued by NIH; (4) studies not comparing the metabolism between PCOS patients with and without HA, or the metabolism among patients with different PCOS phenotypes; (5) studies not involving outcomes; and (6) if the same agency published a number of articles with overlapping time span, earlier studies were excluded while only the latest literature was retained.

### Literature filtering, data extraction, and quality assessment

The articles were filtered, data were extracted, and methodological quality was assessed independently by two investigators. Any discrepancy was resolved by discussion or by a third party until a consensus was reached. Data were extracted according to a predesigned table, including general characteristics, type of studies, subjects, factors, and outcomes, etc. PCOS was diagnosed according to different classifications, where PCO + O + HA, PCO + HA, and HA + O were merged as the HA group, and PCO + O was considered as non-HA group of the PCOS.

The Newcastle–OttawaScalewas [4] used to assess the quality of the cohort studies and case–control studies. Quality assessment criteria recommended by the Agency for Healthcare Research and Quality (AHRQ) [5] was used to assess the quality of cross-sectional studies, of which only the former 10 items were selected, since the 11th item was not suitable for assessing the cross-sectional studies, each of which was scored “yes,” “no,” or “unclear.” Quality was assessed independently by two investigators, and any discrepancy was resolved by discussion or by three other authors in this study.

### Statistical analysis

Meta-analysis was performed using the Stata 12.0 software. Categorical variables were expressed as odds ratio and 95 % confidence interval (95%CI). Continuous variables were expressed as mean difference or standardized mean difference (SMD) and 95%CI. The enrolled articles were tested for heterogeneityusing the*χ* ^*2*^ test, with an inspection level *α* = 0.1 or *P* ≤ 0.1, and the results of various articles were found to be heterogeneous. Heterogeneity was assessed using*I* ^2^, where *I* ^2^ ≥ 25 %, *I* ^2^ ≥ 50 %, and *I* ^2^ ≥ 75 % referred to a low, moderate, and high degree of heterogeneity, respectively. If there was no heterogeneity among various studies, the meta-analyses were performed using a fixed-effect model. Otherwise, meta-analyses were performed using a random effect model, and the source of the heterogeneity was further analyzed and possible factors were performed subgroup analyses, of which description analyses were adopted if there existed excessive heterogeneity between the two groups or it was impossible to find the data resources. A difference with *P* < 0.05 was considered statistically significant. Then, sensitivity analyses were conducted by excluding the impact of individual study one by one on the overall results of the analysis. Moreover, publication bias was quantitatively assessed using the funnel plot and Egger test.

## Results

### Literature search results

**Fig. 1:**
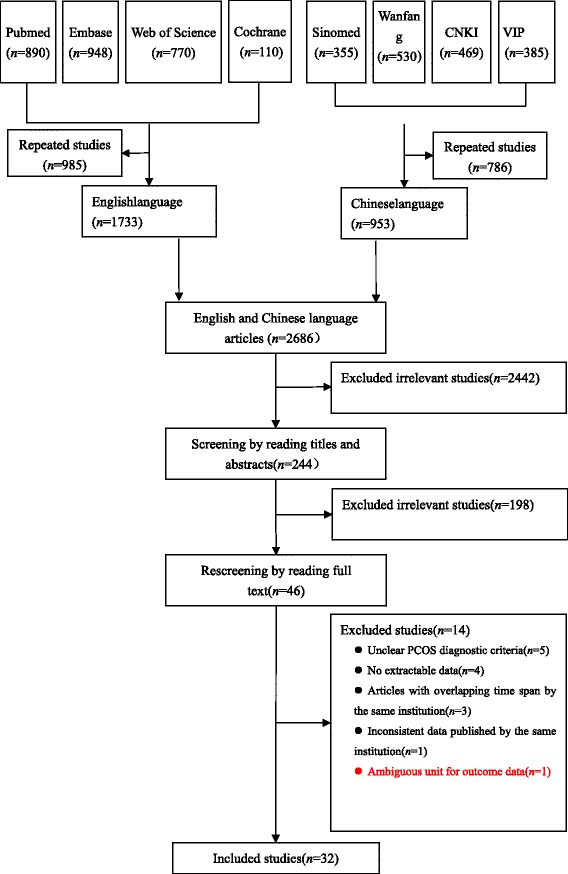
Flow chart demonstrating study selection

A total of 4457 articles were preliminarily searched, and ultimately 32 articles were included after layer-by-layer screening [6–37]. The screening flowchart and results are shown in Fig. 1.

### Characteristics and quality assessment of the included studies

The 32 articles included were cross-sectional studies, involving 9556 patients, of which 6482 cases were in the HA group and 3074 cases in the non-HA group. The basic characteristics and quality assessment of the included studies are shown in Tables 1 and 2.

**Table 1:** Characteristics of included studies

Included studies	Location	Sample size (hyperandrogenemia/nonhyperandrogenemia)	Mean age (range, year)	PCOS diagnostic criteria	Type of study	Extracted index^j^
Hosseinpanah 2014 [6]	Iran	136 (109/27)	33.6 (18 ~ 45)	2003 Rotterdam criteria	Cross-sectional	^b, d, f^
Kim 2014 [7]	Korea	700 (432/268)	27.9 (15 ~ 40)	2003 Rotterdam criteria	Cross-sectional	^a^
Lerchbaum 2014 [8]	Austria	706 (352/354)	27^h^ (16 ~ 45)	2003 Rotterdam criteria	Cross-sectional	^a, b^
Livadas 2014 [9]	Greece	1218 (716/502)	23^h^	2003 Rotterdam criteria	Cross-sectional	–
Sung 2014 [10]	Korea	1062 (645/417)	24	2003 Rotterdam criteria	Cross-sectional	^a, c, d, e, f^
Tehrani 2014 [11]	Iran	85 (72/13)	29.07 (18 ~ 45)	2003 Rotterdam criteria	Cross-sectional	^a, c, d, e, f, g^
Ates 2013 [12]	Turkey	410 (334/76)	24.55	2003 Rotterdam criteria	Cross-sectional	^a, c, e, f, g^
Di Sarra 2013 [13]	Italy	89 (65/24)	23.6 (18 ~ 40)	2003 Rotterdam criteria	Cross-sectional	^d, e, f, g^
Zhu 2013 [14]	Shanghai, China	53 (28/25)	22.82	2003 Rotterdam criteria	Cross-sectional	^d, e, f, g^
Gluszak 2012 [15]	Poland	93 (88/5)	23.95	2003 Rotterdam criteria	Cross-sectional	^c, d, e, f, g^
Jones 2012 [16]	United Kingdom	29 (19/10)	28	2003 Rotterdam criteria	Cross-sectional	–
Li 2012 [17]	Guangdong, China	131 (62/69)	29.57	2003 Rotterdam criteria	Cross-sectional	^c, d, e, f, g^
Ozkaya 2012 [18]	Turkey	132 (100/32)	24.21	2003 Rotterdam criteria	Cross-sectional	^c, d, e, f, g^
Cupisti 2011^i^ [19]	Germany	309 (293/16)	27.16	2006AES criteria^i^	Cross-sectional	^c, d, e, f, g^
Mehrabian 2011 [20]	Iran	539 (287/252)	29.3 (18 ~ 42)	2003 Rotterdam criteria	Cross-sectional	^a, b, c, f^
Melo 2011 [21]	Brazil	226 (175/51)	26.45	2003 Rotterdam criteria	Cross-sectional	^a, c, d, e, f, g^
Wijeyaratne 2011 [22]	Sri Lanka	469 (374/95)	25	2003 Rotterdam criteria	Cross-sectional	^a^
Yilmaz 2011 [23]	Turkey	127 (103/24)	25.36 (18 ~ 35)	2003 Rotterdam criteria	Cross-sectional	^a, c, d, e, f, g^
Castelo-Branco 2010 [24]	Spain	197 (152/45)	28.4	2003 Rotterdam criteria	Cross-sectional	^e, f, g^
Guo 2010 [25]	Shandong, China	615 (571/44)	28.3 (20 ~ 41)	2003 Rotterdam criteria	Cross-sectional	^a, c, d, e, f, g^
Goverde 2009 [26]	Netherlands	157 (101/56)	29 (17 ~ 43)	2003 Rotterdam criteria	Cross-sectional	^a, b, c, f^
Barber 2007 [27]	United Kingdom	309 (267/42)	33.26	2003 Rotterdam criteria	Cross-sectional	^a^
Shroff 2007 [28]	United States	258 (224/34)	27.86 (18 ~ 45)	2003 Rotterdam criteria	Cross-sectional	^a, c, d, e, f, g^
Chen H 2014 [29]	Shanghai, China	126 (34/92)	27	2003 Rotterdam criteria	Cross-sectional	^c, d, e, f, g^
Li YC 2014 [30]	Guangxi, China	68 (42/26)	25.51 (18 ~ 37)	2003 Rotterdam criteria	Cross-sectional	^d, e, f, g^
Ha LX 2013 [31]	Ningxia, China	267 (127/140)	25.21	2003 Rotterdam criteria	Cross-sectional	^c, d, e, f, g^
Tao T 2013 [32]	Shanghai, China	305 (248/57)	26.44 (18 ~ 45)	2003 Rotterdam criteria	Cross-sectional	^a^
Li J 2011 [33]	Shanghai, China	95 (84/11)	Unknown	2003 Rotterdam criteria	Cross-sectional	^c, d, e, f, g^
Liu L 2011 [34]	Zhejiang, China	48 (34/14)	27.15 (23 ~ 33)	2003 Rotterdam criteria	Cross-sectional	^d, e, f, g^
Qu ZY 2011 [35]	Shandong, China	306 (177/129)	Unknown	2003 Rotterdam criteria	Cross-sectional	^b^
Xu LS 2010 [36]	Tianjin, China	256 (152/104)	23.8 (14 ~ 39)	2003 Rotterdam criteria	Cross-sectional	^b, c^
Zhang L 2010 [37]	Jiangsu, China	35 (15/20)	29.43 (21 ~ 35)	2003 Rotterdam criteria	Cross-sectional	^b^

^a^Number of cases with MetS; ^b^Number of cases with IR; ^c^HOMA-IR value; ^d^TC value; ^e^TG value; ^f^HDL value; ^g^LDL value; ^h^Median; ^i^PCOS typing had10 subtypes, and the rest had four subtypes; ^j^Meant that the corresponding outcome data were not exactable if they were data of median or quartiles that could not be converted into mean ± standard deviation

**Table 2:** Methodological quality assessment of the included cross-sectional studies

Included studies	Q1	Q2	Q3	Q4	Q5	Q6	Q7	Q8	Q9	Q10
Hosseinpanah 2014 [6]	Yes	Yes	Yes	Yes	Unclear	Yes	No	No	No	Yes
Kim 2014 [7]	Yes	Yes	Yes	Unclear	Unclear	Yes	No	Yes	No	No
Lerchbaum 2014 [8]	Yes	Yes	Yes	Unclear	Unclear	Yes	No	No	No	Yes
Livadas 2014 [9]	Yes	Yes	Yes	Yes	Unclear	Yes	No	Yes	No	Yes
Sung 2014 [10]	Yes	Yes	Yes	Unclear	Unclear	Yes	No	Yes	No	Yes
Tehrani 2014 [11]	Yes	Yes	Yes	Yes	Unclear	Yes	No	No	No	Yes
Ates 2013 [12]	Yes	Yes	Yes	Unclear	Unclear	Yes	No	No	No	Yes
Di Sarra 2013 [13]	Yes	Yes	No	Unclear	Unclear	No	No	No	No	Yes
Zhu 2013 [14]	Yes	Yes	Yes	Unclear	Unclear	Yes	No	Yes	No	Yes
Gluszak 2012 [15]	No	Yes	No	Unclear	Unclear	No	No	No	No	Yes
Jones 2012 [16]	No	Yes	No	Unclear	Unclear	Yes	No	No	No	Yes
Li 2012 [17]	No	Yes	No	Unclear	Unclear	Yes	No	No	No	Yes
Ozkaya 2012 [18]	Yes	Yes	Yes	Yes	Unclear	Yes	No	No	No	Yes
Cupisti 2011 [19]	No	Yes	Yes	Unclear	Unclear	Yes	No	No	No	No
Mehrabian 2011 [20]	Yes	Yes	Yes	Unclear	Unclear	Yes	No	No	No	Yes
Melo 2011 [21]	Yes	Yes	Yes	Yes	Unclear	Yes	No	No	No	Yes
Wijeyaratne 2011 [22]	Yes	Yes	Yes	Yes	Unclear	Yes	No	No	No	No
Yilmaz 2011 [23]	Yes	Yes	Yes	Unclear	Unclear	No	No	No	No	Yes
Castelo-Branco 2010 [24]	No	Yes	Yes	Yes	Unclear	Yes	No	No	No	Yes
Guo 2010 [25]	Yes	Yes	Yes	Unclear	Unclear	Yes	No	No	No	Yes
Goverde 2009 [26]	Yes	Yes	No	Unclear	Unclear	Yes	No	No	No	No
Barber 2007 [27]	Yes	Yes	No	Unclear	Unclear	Yes	No	No	No	No
Shroff 2007 [28]	Yes	Yes	Yes	Unclear	Unclear	No	No	Yes	No	Yes
Chen H 2014 [29]	Yes	Yes	Yes	Unclear	Unclear	Yes	No	No	No	Yes
Li YC 2014 [30]	Yes	Yes	Yes	Unclear	Unclear	Yes	No	No	No	Yes
Ha LX 2013 [31]	Yes	Yes	Yes	Unclear	Unclear	Yes	No	No	No	Yes
Tao T 2013 [32]	Yes	Yes	Yes	Unclear	Unclear	Yes	No	No	No	Yes
Li J 2011 [33]	Yes	Yes	Yes	Unclear	Unclear	Yes	No	No	No	Yes
Liu L 2011 [34]	Yes	Yes	Yes	Unclear	Unclear	Yes	No	No	No	No
Qu ZY 2011 [35]	Yes	Yes	Yes	Unclear	Unclear	Yes	No	No	No	Yes
Xu LS 2010 [36]	Yes	Yes	Yes	Unclear	Unclear	Yes	No	No	No	Yes
Zhang L 2010 [37]	Yes	Yes	Yes	Unclear	Unclear	Yes	No	No	No	Yes

AHRQ was used to assess the quality of the cross-sectional studies—Q1:whether there was a clear source of data (surveys, literature review);Q2:whether the inclusion and exclusion criteria of the exposure or nonexposure groups (case and control groups) were listed or referred to as previous literature;Q3:whether the period of time to identify patients was provided;Q4:for subjects who did not come from the crowd, whether they were continuously observed;Q5:whether the other aspects of the subjects were overshadowed by the subjective factors of the evaluators;Q6:whether any evaluation to ensure the quality was described (such as test/retest of the primary outcomes);Q7:whether the reasons to exclude any patient were provided;Q8:whether the measures to evaluate and control confounding factors were described;Q9:if possible, whether the studies explain how to handle the missing data;Q10:whether the studies summarized the response rate of the patients and the integrity of data collection

### Meta-analysis results

#### Incidence of metabolic syndrome

**Fig. 2:**
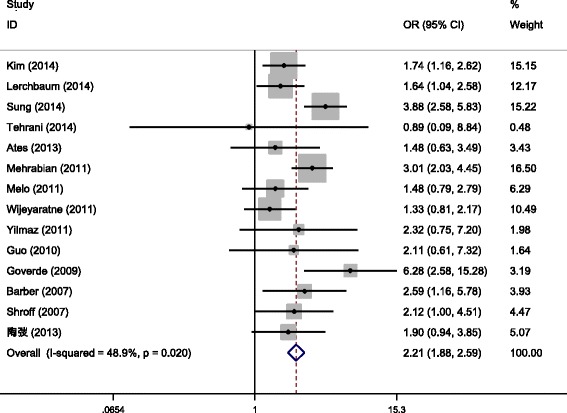
Meta-analysis for the effects of HAon the incidence of MetS in PCOS patients

Among the enrolled articles, the incidence of MetS was involved in 14 studies [a total of 5968 PCOS patients, including 4185 cases in the PCOS patients with HA (PCOS/HA) group and 1783 cases in the PCOS patients without HA (PCOS/NHA group)] [8, 9, 11–13, 21–24, 26–29, 33]. Since results of different studies were heterogeneous (*P* = 0.020, *I* ^2^ = 48.9 %), OR was combined using Peto method for meta-analysis, and the results revealed that the incidence of MetS showed statistical significance between the PCOS/HA and the PCOS/NHA groups [Peto OR = 2.21, 95 % CI(1.88,2.59), *P* < 0.001 (Fig. 2)]. Then sensitivity analyses were performed after excluding one study with large heterogeneity, and the results revealed that the combined effect quantity was still of statistical significance and no changes occurred in the forest map structure.

#### HOMA-IR

**Fig. 3:**
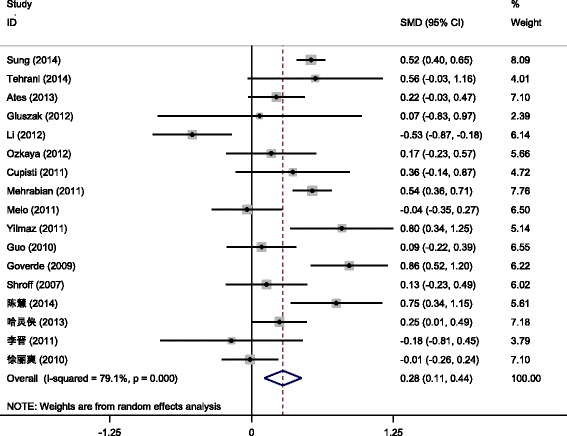
Meta-analysis for effects of HAon HOMA-IR in PCOS patients

HOMA-IR was mentioned in 17 out of the included articles [11–13, 16, 18–22, 24, 26, 27, 29, 30, 32, 34, 37] (a total of 4888 PCOS patients, including 3452 cases in the PCOS/HA group and 1436 cases in the PCOS/NHA group). Since results of different studies were heterogeneous (*P* < 0.001, *I* ^2^ = 79.1 %), the random effect model was used for meta-analysis and the results showed that the difference of HOMA-IR was statistically significant between the PCOS/HA and PCOS/NHA groups [SMD = 0.28, 95 % CI (0.11,0.44), *P* = 0.001 (Fig. 3)].

#### Incidence of insulin resistance

**Fig. 4:**
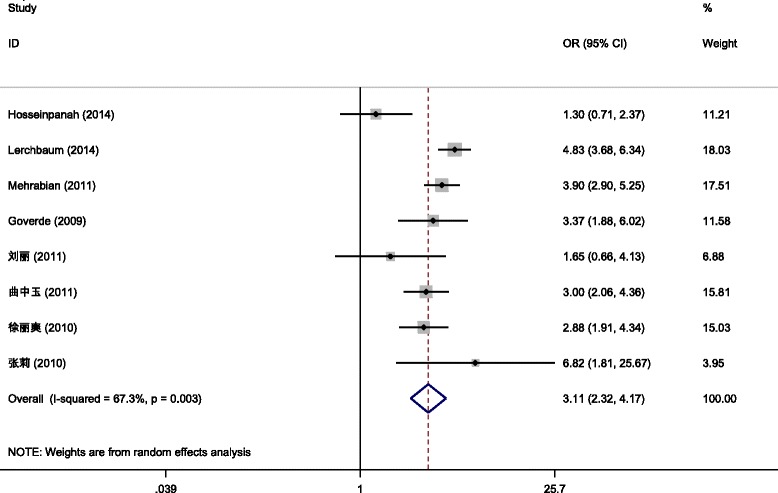
Meta-analysis for the effects of HA on the incidence of IR in PCOS patients

Incidence of IR was involved in eight [7, 9, 21, 27, 35–37] out of the included articles (a total of 2183 patients, including 1227 cases in the PCOS/HA group and 956 cases in the PCOS/NHA group). Since results of different studies were heterogeneous (*P* = 0.003, *I* ^2^ = 67.3 %), the random effect model was adopted for meta-analysis, and the results revealed that the incidence of IR was statistically significant between the PCOS/HA and PCOS/NHA groups [OR = 3.11, 95 % CI(2.32,4.17), *P* < 0.001 (Fig. 4)].

#### Lipid metabolism

Lipid metabolism indexes included total cholesterol (TC), triglycerides (TG), high-density lipoprotein (HDL), and low-density lipoprotein (LDL).

1. *Total cholesterol*
  TC was involved in 18 [7, 11, 12, 14–16, 18–20, 22, 24–26, 29–32, 34, 35] out of the included articles (a total of 3920 PCOS patients, including 2856 cases in the PCOS/HA group and 1064 cases in the PCOS/NHA group). Meta-analysis was performed using the random effect model due to heterogeneity among different studies (*P* = 0.002, *I* ^2^ = 56.6 %), and the results showed that the difference of TC was not statistically significant between the PCOS/HA and PCOS/NHA groups [SMD = 0.05, 95 % CI (−0.09,0.18), *P* = 0.494].
2. *Triglycerides*
  TG was involved in 19 [11–16, 18–20, 22, 24–26, 29–32, 34, 35] out of the included articles (a total of 4391 PCOS patients, including 3233 cases in the PCOS/HA group and 1158 cases in the PCOS/NHA group). Meta-analysis was conducted using the random effect model due to heterogeneity among different studies (*P* < 0.001, *I* ^2^ = 72.7 %), which revealed that the difference of TG was statistically insignificant between the PCOS/HA and PCOS/NHA groups [SMD = 0.15, 95 % CI (−0.01, 0.31), *P* = 0.061].
3. *High-density lipoprotein*
  HDL was involved in 22 [7, 11–16, 18–22, 24–27, 29–32, 34, 35] out of the included articles (a total of 5223 PCOS patients, including 3730 cases in the PCOS/HA group and 1493 cases in the PCOS/NHA group). Also, meta-analysis was conducted using the random effect model due to heterogeneity among different studies (*P* < 0.001, *I* ^2^ = 80.9 %), which showed that the difference of HDL was statistically significant between the PCOS/HA and PCOS/NHA groups [SMD = -0.22, 95 % CI (-0.39,-0.06), *P* = 0.009].
4. *Low-density lipoprotein*
  LDL was mentioned in 18 [12–16, 18–20, 22, 24–26, 29–32, 34, 35] out of the included articles (a total of 3329 PCOS patients, including 2588 cases in the PCOS/HA group and 741 cases in the PCOS/NHA group). Again, meta-analysis was conducted using the random effect model due to heterogeneity among different studies (*P* < 0.001, *I* ^2^ = 66.0 %), which revealed that the difference of LDL was statistically insignificant between the PCOS/HA and PCOS/NHA groups [SMD = 0.14, 95 % CI (−0.03,0.30), *P* = 0.106].

### Publication bias

**Fig. 5:**
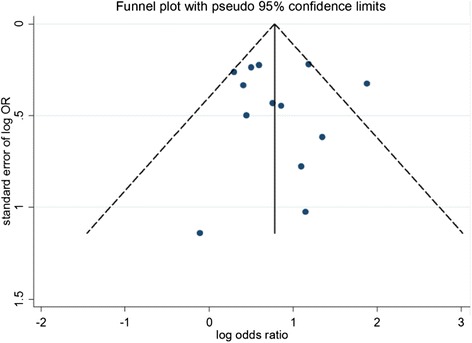
Funnel Plot analysis of publication bias of impact of hyperandrogenemia on the incidence of metabolic syndrome in PCOS patients

Publication bias was analyzed using the funnel plot, and the results revealed a good symmetric distribution of the included studies on both sides of the funnel plot, suggesting a small possibility of publication bias. Also, publication bias was not found in further Egger test (*P* = 0.820) (Fig. 5).

## Discussions

A total of 32 articles were included in this systematic assessment, and the meta-analysis revealed that the incidence of MetS, HOMA-IR value, incidence of IR were higher in the PCOS/HA group compared with the PCOS/NHA group, and the HDL value in the PCOS/HA group was smaller than that in the PCOS/NHA group, while TC, TG, and LDL were not significantly different between the PCOS/HA and PCOS/NHA groups. The included 32 articles were cross-sectional studies, with a large sample size and ordinary quality. Sensitivity and publication bias analyses showed stable meta-analysis results, while there existed a large heterogeneity among the studies, which might affect the results.

Limitations of this study included the following: (1) All the included articles were cross-sectional studies, and there was a lack of cohort studies and case–control studies, presenting a low argumentation intensity. (2) All the included articles were published literature, and there was a lack of gray literature, which might lead to publication bias. (3) This study failed to conduct subgroup analyses in patients from different regions, hereby the bias caused by population factors from different regions could not be excluded. (4) In most of the included studies, important confounding factors such as age, body mass index (BMI), waistline and waist–hipratio, etc., were not adjusted. However, metabolic abnormalities might be different among patients with different ages, BMIs, waistlines, and waist–hipratios, which are prone to affect the meta-analysis results. (5) There was a large heterogeneity among studies, which might affect the meta-analysis results.

## Conclusions

HA play a role between PCOS and MetS. There were differences in HOMA-IR and IR incidence between PCOS/HA and PCOS/NHA; also the lipid metabolism might present a trend of variation between PCOS/HA and PCOS/NHA patients. However, due to the limitations of sample size and quality, the present-study conclusions require further verification using a larger sample size and high-quality studies.

## Abbreviations

HA: Hyperandrogenism
HDL: High density lipoprotein
HOMA-IR: Homeostasis model assessment insulin resistance index
LDL: Low density lipoprotein
MetS: Metabolic syndrome
PCOS: Polycystic ovary syndrome
TC: Total cholesterol
TG: Triglyceride

## References

[1] Dunaif A (1995). Hyperandrogenic anovulation (PCOS): a unique disorder of insulin action associated with an increased risk of non-insulin-dependent diabetes mellitus. Am J Med.

[2] Park HR, Choi Y, Lee HJ, Oh JY, Hong YS, Sung YA (2007). The metabolic syndrome in young Korean women with polycystic ovary syndrome. Diabetes Res Clin Pract.

[3] Teede H, Deeks A, Moran L (2010). Polycystic ovary syndrome: a complex condition with psychological, reproductive and metabolic manifestations that impacts on health across the lifespan. BMC Med.

[4] Wells GA, Shea B, O’Connell D, Peterson J, Welch V, Losos M, Tugwell P (2013). The Newcastle-Ottawa Scale (NOS) for assessing the quality of nonrandomized studies in meta-analyses. PLoS Negl Trop Dis.

[5] Rostom A, Dubé C, Cranney A, Saloojee N, Sy R, Garritty C, Sampson M, Zhang L, Yazdi F, Mamaladze V, Pan I, McNeil J, Moher D, Mack D, Patel D (2004). Celiac disease.

[6] Hosseinpanah F, Barzin M, Keihani S, Ramezani Tehrani F, Azizi F (2014). Metabolic aspects of different phenotypes of polycystic ovary syndrome: Iranian PCOS Prevalence Study. Clin Endocrinol (Oxf).

[7] Kim MJ, Lim NK, Choi YM, Kim JJ, Hwang KR, Chae SJ, Park CW, Choi DS, Kang BM, Lee BS, Kim T, Park HY (2014). Prevalence of metabolic syndrome is higher among non-obese PCOS women with hyperandrogenism and menstrual irregularity in Korea. PLoS One.

[8] Lerchbaum E, Schwetz V, Rabe T, Giuliani A, Obermayer-Pietsch B (2014). Hyperandrogenemia in polycystic ovary syndrome: exploration of the role of free testosterone and androstenedione in metabolic phenotype. PLoS One.

[9] Livadas S, Pappas C, Karachalios A, Marinakis E, Tolia N, Drakou M, Kaldrymides P, Panidis D, Diamanti-Kandarakis E (2014). Prevalence and impact of hyperandrogenemia in 1,218 women with polycystic ovary syndrome. Endocrine.

[10] Sung YA, Oh JY, Chung H, Lee H (2014). Hyperandrogenemia is implicated in both the metabolic and reproductive morbidities of polycystic ovary syndrome. Fertil Steril.

[11] Tehrani FR, Rashidi H, Khomami MB, Tohidi M, Azizi F (2014). The prevalence of metabolic disorders in various phenotypes of polycystic ovary syndrome: a community based study in Southwest of Iran. Reprod Biol Endocrinol.

[12] Ates S, Sevket O, Sudolmus S, Dane B, Ozkal F, Uysal O, Dansuk R (2013). Different phenotypes of polycystic ovary syndrome in Turkish women: clinical and endocrine characteristics. Gynecol Endocrinol.

[13] Di Sarra D, Tosi F, Bonin C, Fiers T, Kaufman JM, Signori C, Zambotti F, Dall’Alda M, Caruso B, Zanolin ME, Bonora E, Moghetti P (2013). Metabolic inflexibility is a feature of women with polycystic ovary syndrome and is associated with both insulin resistance and hyperandrogenism. J Clin Endocrinol Metab.

[14] Zhu JP, Teng YC, Zhou J, Lu W, Tao MF, Jia WP (2013). Increased mean glucose levels in patients with polycystic ovary syndrome and hyperandrogenemia as determined by continuous glucose monitoring. Acta Obstet Gynecol Scand.

[15] Gluszak O, Stopinska-Gluszak U, Glinicki P, Kapuscinska R, Snochowska H, Zgliczynski W, Debski R (2012). Phenotype and metabolic disorders in polycystic ovary syndrome. ISRN Endocrinol.

[16] Jones H, Sprung VS, Pugh CJ, Daousi C, Irwin A, Aziz N, Adams VL, Thomas EL, Bell JD, Kemp GJ, Cuthbertson DJ (2012). Polycystic ovary syndrome with hyperandrogenism is characterized by an increased risk of hepatic steatosis compared to nonhyperandrogenic PCOS phenotypes and healthy controls, independent of obesity and insulin resistance. J Clin Endocrinol Metab.

[17] Li Y, Ma Y, Chen X, Wang W, Li Y, Zhang Q, Yang D (2012). Different diagnostic power of anti-Mullerian hormone in evaluating women with polycystic ovaries with and without hyperandrogenism. J Assist Reprod Genet.

[18] Ozkaya E, Cakir E, Cinar M, Kara F, Baser E, Cakir C, Kucukozkan T (2012). Is hyperandrogenemia protective for fibrocystic breast disease in PCOS?. Gynecol Endocrinol.

[19] Cupisti S, Haeberle L, Schell C, Richter H, Schulze C, Hildebrandt T, Oppelt PG, Beckmann MW, Dittrich R, Mueller A (2011). The different phenotypes of polycystic ovary syndrome: no advantages for identifying women with aggravated insulin resistance or impaired lipids. Exp Clin Endocrinol Diabetes.

[20] Mehrabian F, Khani B, Kelishadi R, Kermani N (2011). The prevalence of metabolic syndrome and insulin resistance according to the phenotypic subgroups of polycystic ovary syndrome in a representative sample of Iranian females. J Res Med Sci.

[21] Melo AS, Vieira CS, Romano LG, Ferriani RA, Navarro PA (2011). The frequency of metabolic syndrome is higher among PCOS Brazilian women with menstrual irregularity plus hyperandrogenism. Reprod Sci.

[22] Wijeyaratne CN, Seneviratne Rde A, Dahanayake S, Kumarapeli V, Palipane E, Kuruppu N, Yapa C, Seneviratne Rde A, Balen AH (2011). Phenotype and metabolic profile of South Asian women with polycystic ovary syndrome (PCOS): results of a large database from a specialist Endocrine Clinic. Hum Reprod.

[23] Yilmaz M, Isaoglu U, Delibas IB, Kadanali S (2011). Anthropometric, clinical and laboratory comparison of four phenotypes of polycystic ovary syndrome based on Rotterdam criteria. J Obstet Gynaecol Res.

[24] Castelo-Branco C, Steinvarcel F, Osorio A, Ros C, Balasch J (2010). Atherogenic metabolic profile in PCOS patients: role of obesity and hyperandrogenism. Gynecol Endocrinol.

[25] Guo M, Chen ZJ, Macklon NS, Shi YH, Westerveld HE, Eijkemans MJ, Fauser BC, Goverde AJ (2010). Cardiovascular and metabolic characteristics of infertile Chinese women with PCOS diagnosed according to the Rotterdam consensus criteria. Reprod Biomed Online.

[26] Goverde AJ, van Koert AJ, Eijkemans MJ, Knauff EA, Westerveld HE, Fauser BC, Broekmans FJ (2009). Indicators for metabolic disturbances in anovulatory women with polycystic ovary syndrome diagnosed according to the Rotterdam consensus criteria. Hum Reprod.

[27] Barber TM, Wass JA, McCarthy MI, Franks S (2007). Metabolic characteristics of women with polycystic ovaries and oligo-amenorrhoea but normal androgen levels: implications for the management of polycystic ovary syndrome. Clin Endocrinol (Oxf).

[28] Shroff R, Syrop CH, Davis W, Van Voorhis BJ, Dokras A (2007). Risk of metabolic complications in the new PCOS phenotypes based on the Rotterdam criteria. Fertil Steril.

[29] Chen H, Yang ZF, Zhan WW, La DD, Chen C. Ultrasonographic features of polycystic ovary syndrome and their correlation with endocrinology index. J Diagn Concepts Pract. 2014;13(2):171–5.

[30] Li YC (2014). Relationship of lipid metabolism disturbance with body weight, insulin resistance and androgen level in patients with polycystic ovary syndrome. Matern Child Health Care China.

[31] Ha LX, Shi YH, Zhao JL, Chen ZJ (2013). Lipid metabolism characteristics in polycystic ovary syndrome patientswith different androgen levelsin Ningxia. J Shandong University (Med Sci).

[32] Tao T, Liu W, Zhao AM, Li SX, Zheng J, Wang LH, Zhou JW, Huang R, Zhang P (2013). Characteristics and risk of metabolic syndrome in patients with different phenotypes of polycystic ovary syndrome. Chin J Endocrinol Metab.

[33] Li J, Xu C, Zhang HJ, Hong J, Ning G, Li XY (2011). Correlation of obesity with hyperandrogenism and insulin resistance in patients with polycystic ovary syndrome. Chin J Endocrinol Metab.

[34] Liu L, Shao JB, Zhang HP, Shen XL (2011). Metabolism characteristics in patients with different phenotypes of polycystic ovary syndrome. Clin Educ Gen Pract.

[35] Qu ZY, Shi YH, Chen ZJ (2011). Relationship between hyperandrogenism and non-alcoholic fatty liver disease of polycystic ovary syndrome. Reprod Contracept.

[36] Xu LS, Zhang YF, Zhang HY, Xue FX, Han YK (2010). Glucose metabolism characteristics in patients with different phenotypes of polycystic ovary syndrome. Prog Obstet Gynecol.

[37] Zhang L, Zhang Y (2010). Correlation of androgen level with insulin resistance in patients with polycystic ovary syndrome. Exp Lab Med.

